# Signatures of Adaptation in Human Invasive *Salmonella* Typhimurium ST313 Populations from Sub-Saharan Africa

**DOI:** 10.1371/journal.pntd.0003611

**Published:** 2015-03-24

**Authors:** Chinyere K. Okoro, Lars Barquist, Thomas R. Connor, Simon R. Harris, Simon Clare, Mark P. Stevens, Mark J. Arends, Christine Hale, Leanne Kane, Derek J. Pickard, Jennifer Hill, Katherine Harcourt, Julian Parkhill, Gordon Dougan, Robert A. Kingsley

**Affiliations:** 1 Wellcome Trust Sanger Institute, Wellcome Trust Genome Campus, Hinxton, Cambridge, United Kingdom; 2 Institute for Molecular Infection Biology, University of Wurzburg, Wuerzburg, Germany; 3 Cardiff School of Biosciences, Cardiff University, Cardiff, United Kingdom; 4 The Roslin Institute & Royal (Dick) School of Veterinary Studies, University of Edinburgh, Easter Bush, Midlothian, Scotland, United Kingdom; 5 Department of Pathology, University of Cambridge, Addenbrokes Hospital, Cambridge, United Kingdom; 6 Edinburgh Cancer Research Centre, University of Edinburgh, Western General Hospital, Edinburgh, United Kingdom; 7 Institute of Food Research, Norwich Research Park, Norwich, Norfolk, United Kingdom; Beijing Institute of Microbiology and Epidemiology, CHINA

## Abstract

Two lineages of *Salmonella enterica* serovar Typhimurium (*S*. Typhimurium) of multi-locus sequence type ST313 have been linked with the emergence of invasive *Salmonella* disease across sub-Saharan Africa. The expansion of these lineages has a temporal association with the HIV pandemic and antibiotic usage. We analysed the whole genome sequence of 129 ST313 isolates representative of the two lineages and found evidence of lineage-specific genome degradation, with some similarities to that observed in *S*. Typhi. Individual ST313 *S*. Typhimurium isolates exhibit a distinct metabolic signature and modified enteropathogenesis in both a murine and cattle model of colitis, compared to *S*. Typhimurium outside of the ST313 lineages. These data define phenotypes that distinguish ST313 isolates from other *S*. Typhimurium and may represent adaptation to a distinct pathogenesis and lifestyle linked to an-immuno-compromised human population.

## Author Summary

*Salmonella enterica* is a diverse species, isolates of which can colonise or infect many different animals, including humans and can cause different disease syndromes. *S*. *enterica* can be sub-typed using serology into serovars. Isolates from some serovars, known as generalists, can infect multiple hosts (e.g. *S*. Typhimurium) and usually cause gastroenteritis. However, other serovars exhibit host adaptation or even restriction. Host-adapted serovars such as *S*. Dublin show preference for a particular host but can also infect other hosts, while host-restricted serovars are capable of infecting only a single host (e.g. *S*. Typhi in humans) and frequently cause febrile systemic disease (typhoid). In this study, we use genotypic and phenotypic methods to investigate clinical isolates representative of populations of two recently emerged *S*. Typhimurium lineages of type ST313 associated with invasive disease in sub-Saharan Africa. Our results identify potential characteristics in these isolates that may be associated with adaptation to invasive disease in humans with a compromised immunity.

## Introduction

*Salmonella enterica* isolates can infect a range of animals and humans, causing a spectrum of disease syndromes ranging from gastroenteritis through to typhoid and an asymptomatic carrier state [1]. From a clinical perspective *S*. *enterica* serovars have been classically assigned to two broad groups, typhoidal or non-typhoidal *Salmonella* (NTS). Typhoidal *Salmonella* include the human restricted *S*. *enterica* serovar Typhi (*S*. Typhi), the cause of the systemic disease typhoid fever, which is strictly transmitted within the human population independently of a zoonotic reservoir. NTS, on the other hand, are predominantly associated with self-limiting gastroenteritis, largely originating from zoonotic reservoirs with human-to-human transmission regarded as being relatively rare [2].

Invasive NTS (iNTS) disease in sub-Saharan Africa does not fit well into the classical view of salmonellosis. NTS has emerged as a significant cause of invasive human disease, exceeding *S*. Typhi in many parts of the region as the leading cause of invasive salmonellosis. Humans can be predisposed to this disease by immune suppression or co-infections, which include severe malaria in children and HIV in adults [3,4]. Invasive NTS clinical syndrome is somewhat dissimilar to both typhoid fever and gastroenteritis, and includes non-specific fever and only sporadic or limited diarrhea [5]. High case fatalities have been reported in children and adults in the absence of adequate treatment [6–9].

We recently reported that the emergence of iNTS disease within the sub-Saharan region has been associated with the emergence of two closely-related, multi-antibiotic resistant lineages of *S*. Typhimurium that belong to multilocus sequence type (MLST) ST313 [10]. Phylogenetic analysis indicated that these ST313 lineages emerged independently in recent decades, in close temporal association with the HIV pandemic [10]. As no obvious zoonotic source of ST313 *S*. Typhimurium has been identified, it has been postulated that these lineages may be undergoing host adaptation to humans and may be transmitted, at least in part, directly from human-to-human [11]. Additionally, emergence of the lineages was concomitant with acquisition of multidrug resistance (MDR) including chloramphenicol in one lineage.

Genome sequencing of a representative ST313 isolate, D23580 from Malawi, identified distinct genetic signatures not present in other sequenced non-ST313 *S*. Typhimurium [5]. For example, the genome of D23580 exhibited considerable genome degradation with some similarity to that observed in *S*. Typhi [5]. Genome degradation, in the form of the accumulation of so-called pseudogenes, is a signature of some host restricted pathogens including *Bordetella pertussis* [12,13], *S*. Typhi [14,15], *S*. Paratyphi [15,16] and *S*. Gallinarum [16]. Here, a population-based approach was used to assess how genome degradation emerged within the ST313 lineages. In addition, we used a range of approaches to phenotype representatives of the ST313 in an effort to link the genotypic differences to metabolic and virulence-associated phenotypic differences.

## Results

### Genome degradation is evident across the *S*. Typhimurium ST313 lineages

**Fig 1:**
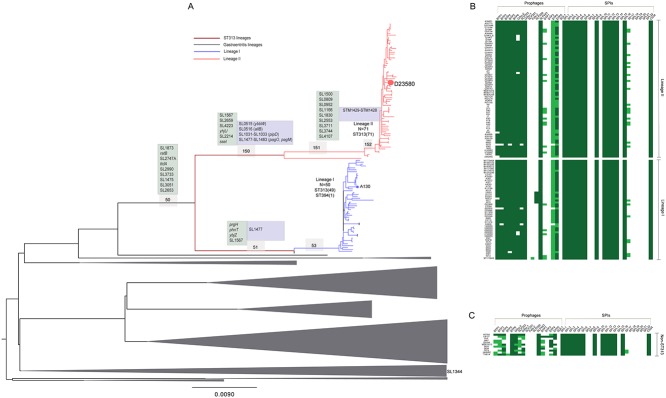
Characterisation and distribution of SNPs in ST313 lineage I and lineage II. **A**. Unrooted maximum likelihood tree showing relationships between lineage I (blue), lineage II (red) and gastroenteritis-associated isolates (grey polygons). Size of polygons represents numbers of taxa in the clade. Representative isolates from each group are highlighted within each clade. Scale bar indicates substitutions per variable site. Numbers in light grey boxes indicate parental branches leading to the respective ST313 lineages as shown in Table 1. Text boxes indicate selected degraded genes (pseudogenes—light green and deletions—light blue occurring on the respective branches). Re-used with permission from Okoro *et al*., Nat Genet. 2012. 44(11): 1215–21. doi: 10.1038/ng.2423. List of degraded complement for strain D23580 is adapted from Kingsley *et al*., 2009. **B. Distribution of prophage elements and *Salmonella* pathogenicity islands in ST313 lineage I and II**. Top left panel represents concatenated phage sequences from strains D23580 (‘BTP’), SL1344 (‘SL’) and DT104 (‘DT’). Top right panel represents concatenated sequences of coding and non-coding sequences of SPI-1 to SPI-22 and CSS4 island. Sequence reads mapping to the complete feature length is represented as a heatmap. Green colour indicates >90% (high) coverage; light green indicates >30% but <90% coverage; and white indicates <30% (low) coverage. Isolate order in lineages I and II on left hand panel although not to scale, is according to phylogenetic positioning in 1A. **C** Distribution of phages and SPIs in selected non-ST313 isolates.

ST313 isolates fall into two closely related phylogenetic lineages that are distinct from other *S*. Typhimurium (Fig. 1A, S1 Fig, S1 Table) [10]. Previous genome sequence analysis of one ST313 isolate, D23580 from lineage II, revealed both gene acquisition (e.g. novel phage elements) and genome degradation (e.g. deletions and pseudogenes) in comparison to *S*. Typhimurium in other lineages [5]. To ascertain genome variation within the overall ST313 population, we analysed whole genome sequences of 129 ST313 isolates using the ST19 *S*. Typhimurium SL1344 genome as a reference.

**Table 1:** Genetic variation detected in identified human invasive *S*. Typhimurium lineages.

Variation type	Node	SNPS (Total)	Synonymous	Non-synonymous	Nonsense	Intergenic	Homoplasic	dN/dS	Mean±s.d
Conserved Variation									
Lineage I & II	50	126	51 (40.5%)	51 (40.5%)	4 (3.2%)	20 (15.9%)	22(17.5%)	0.32	
Lineage I	51	12	3 (25.0%)	4 (33.3%)	0 (0.0%)	5 (41.7%)	5(41.7%)	0.43	
	53	215	74 (34.4%)	99 (46.0%)	3 (1.4%)	39 (18.1%)	12 (5.6%)	0.43	
Lineage II	150	127	36 (28.3%)	72 (56.7%)	2 (1.6%)	17 (13.4%)	5 (3.9%)	0.65	
	151	88	32 (36.4%)	37 (42.0%)	1 (1.1%)	18 (20.5%)	2 (2.3%)	0.37	
	152	54	7 (13.0%)	6 (11.1%)	0 (0.0%)	41 (75.9%)	3 (5.6%)	0.28	0.41±0.13
Most recent variation									
Lineage I		335	106 (31.6%)	144 (43.0%)	13 (3.9%)	72 (21.5%)	35 (10.4%)	0.35	
Lineage II		316	84 (26.6%)	159 (50.3%)	8 (2.5%)	64 (20.3%)	28 (8.9%)	0.34	0.35 ±0.007

The frequencies of mutation are in two groups. Percentages give relative frequency of SNP classes within each group and the two ST313 lineages. Last two columns give the mean dN/dS for lineage and standard deviation (s.d.) from the mean.

**Fig 2:**
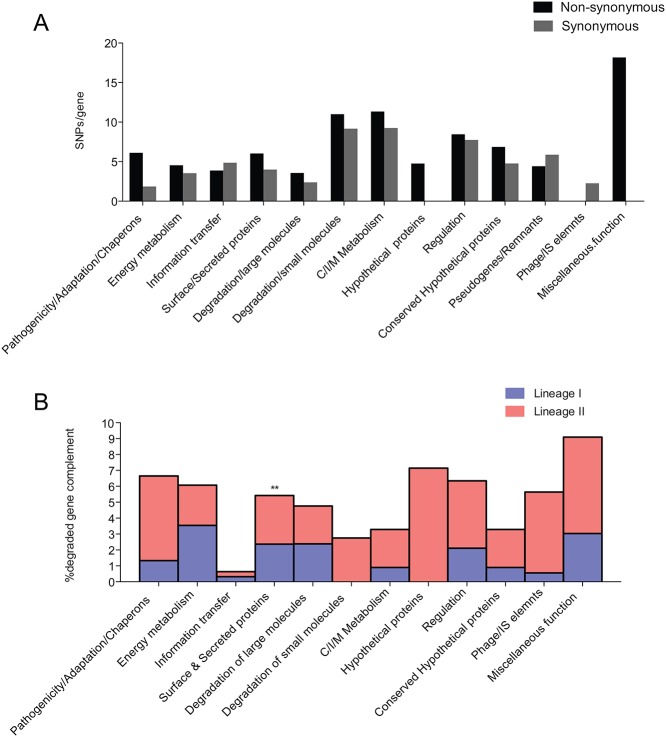
Functional characterisation of SNPs, pseudogenes and small indels in ST313 populations. A. Functional characterisation of non-synonymous (black bars) and synonymous (grey bars) of SNPs in lineages I and II. x-axis shows functional categories used for SNP characterisations; y-axis shows proportion of mutated genes (%) to the no of genes belonging to the functional categories. **B**. Functional characterisation of degraded gene complement (pseudogenes and deletions) in lineage I (blue bars) and lineage II (red bars). x-axis represents % proportion of pseudogenes in each functional group. Asterisks indicate significantly over-represented group with *p* < 0.05 using a two-way ANOVA with post-tests performed using the Bonferroni method.

We first identified the synonymous and non-synonymous single nucleotide polymorphisms (SNPs) in the *S*. Typhimurium ST313 lineages compared to the reference (Fig. 1A, Table 1). The dN/dS ratios for the parental branch of ST313, and that of each lineage since divergence from the last common ancestor, were similar (0.41 ± 0.13 s.d and 0.35 ± 0.007 s.d, respectively, Table 1). Thus, the dN/dS values were smaller than one but still elevated and similar to those expected for recently evolved lineages where time has been too short for purifying selection to act to a significant level [17,18]. The relatively high proportion of non-synonymous SNPs in the two lineages may also represent segregating polymorphisms rather than fixed mutations. Many of these SNPs, both synonymous and non-synonymous, were in metabolic genes and genes involved in degradation of small molecules in both lineages, when compared with SL1344 (Table 1, Fig. 2A). A proportion of the SNPs were also found in genes with no assigned function.

To further characterise the acquisition or loss of genetic material by the ST313 lineages we analysed the whole genome sequence of the additional sequenced isolates. There was little variation in the arrangement of genes within the major virulence-associated *Salmonella* pathogenicity islands (SPIs), including SPIs -1 to -6, -9, -11 to -14, and -16 (Fig. 1B). The previously described prophage elements BTP1, BTP3, BTP4 and BTP6 [5] were present in all isolates and putative deletion events have led to the loss of the phage remnants SLP281 and Fels2 compared to *S*. Typhimurium SL1344 and other non-ST313 isolates included in the analyses. Sequences with similarity to the phage SLP289 were found in a subset of lineage I isolates from Uganda and Kenya but were absent from the rest of the ST313 population. Whole or partial sequences of the *S*. Typhimurium DT104-associated prophage 5 (Fig. 1B) were present in both ST313 lineages, although there was no obvious pattern to the distribution of particular rearrangements of this phage within the ST313 tree. Thus, these data catalogue the major insertions and potential deletions that have occurred since the divergence of ST313 lineages from the last common ancestor.

### Pseudogene formation common to both ST313 lineages

The ST313 sequences were next analysed for evidence of pseudogene formation arising from nonsense SNPs and frame-shift mutation caused by insertions or deletions (<20bp) impacting on all ST313 *S*. Typhimurium used in our analyses. Ten pseudogenes were present in all ST313 isolates but intact in SL1344, including the genes *ttdA*, *ratB* and SL1567 (Fig. 1A, S3 Table). *ttdA* encodes L(+)-tartrate dehydratase, involved in glyoxylate and dicarboxylate metabolism [19]. The gene *ttdA* is also a pseudogene in *S*. Typhi and *S*. Paratyphi A. The *ratB* gene which encodes an outer membrane protein implicated in intestinal persistence in a murine model, is also a pseudogene in *S*. Typhi, *S*. Paratyphi A, *S*. Paratyphi B and the fowl-restricted *S*. Gallinarum [5,20,21]. Other pseudogenes found in all sequenced ST313 include SL1567, a membrane associated protein with different independently acquired nonsense SNPs in lineage I and II, SL2747A, a putative exported protein which may be involved in phospholipid biosynthesis and a transposase, SL1873 (S3 Table). Five pseudogenes were a result of frame-shift mutations (Fig. 1A, S3 Table). These affected the genes SL2990, SL3733, SL1475, SL13051, SL2653, which are predicted to be involved in transcriptional regulation, metabolism and transport and annotated as possessing conserved hypothetical functions, respectively. Most of these genomic signatures represent degradation that occurred before divergence from the last common ancestor of both ST313 lineages.

### Further lineage-specific degradation across the ST313 lineage

In addition to shared genome degradation, lineage-specific nonsense SNPs, frame-shift mutations and small deletions are also present in genes of isolates from ST313 lineages I or II. These represent degradation that occurred after divergence of the two ST313 lineages. Three additional candidate pseudogenes were found in all lineage I isolates. These were in *prfH*, a peptide chain release factor, *phnT*, a probable ATP-binding component of 2-aminoethylphosphonate transporter and *ybjZ* a putative ABC transporter (Fig. 1A, S3 Table); Lineage I isolates also harbour a 700 bp partial deletion within a putative phage gene SL1477. In lineage II, three candidate pseudogenes were a consequence of nonsense SNPs while there was an insertion within SL2214, a putative phage protein in an O-antigen modification locus. The lineage II candidate pseudogenes arising from nonsense SNPs include the gene encoding a conserved hypothetical protein, SL2659 and the membrane proteins *yhjU* and SL4223. The *sseI* gene, which encodes a type III effector, is inactivated by an IS200 element in all lineage II but not lineage I isolates in our collection. Genes associated with allantoin metabolism or transport e.g. *allB*, *gcl*, *glp* and *ybbW* are likely pseudogenes in lineage II. Interestingly genes associated with allantoin metabolism are also inactivated in *S*. Typhi, *S*. Paratyphi A and *S*. Gallinarum. All lineage II isolates possess a partial deletion of the *pipD* gene, encoding a SPI-5 associated protein implicated in persistence in murine macrophages and fluid secretion in bovine models [22,23] (S2 Table). The *phoP/phoQ* regulated genes, *pagO* and *pagM* harbour deletions in all lineage II isolates (Fig. 1A, S2 Table). It is important to note that a number of *phoP/phoQ*-regulated genes are associated with virulence [24] and *pagO* has been previously linked to virulence in porcine models [25]. Additionally, a 4.2kb region encoding plasmid stability proteins was also deleted in all lineage II isolates (S2 Table). There is a statistically significant over-representation of surface/membrane associated and exported proteins inactivated in both lineages (p value <0.05) (Fig. 2B).

### *S*. Typhimurium ST313 isolates exhibit a distinct metabolic profile

**Fig 3:**
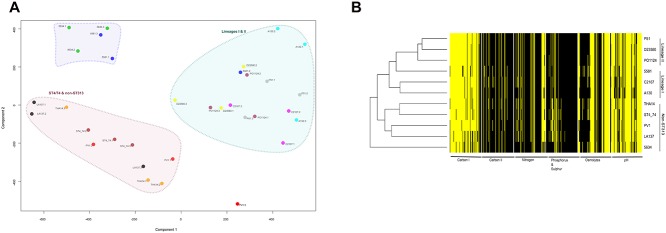
Metabolic profile of ST313 lineage I and lineage II isolates. **A**. Principal component analyses of experiments on isolates and replicates used for 576 metabolic and physiological tests. The axes represent the two principal components (components 1 and 2) that explain the largest amounts of variation observed. Large coloured polygons represent identified clusters as labelled. Replicates of isolates sharing the same polygon colour are labelled with the name of isolates with number suffixes indicating replicate numbers. **B**. Representation of active (yellow) and non-active (black) wells. Varying shades of grey represent ambiguous (possibly active) results.

A systematic analysis of 576 metabolic activities was performed using Biolog phenotype microarrays (PM)[26] on three representatives each of the two ST313 lineages and four *S*. Typhimurium ST19 isolates including SL1344 that acted as experimental controls and comparators (S1 Table). A principal component analysis (PCA) (Fig. 3A) and a hierarchical clustering of Biolog signal values (Fig. 3B) were employed to assess the data sets (S1 Text, S2 Fig). The results from both analyses support the conclusion that ST313 isolates share similar metabolic capacity distinct from ST19 *S*. Typhimurium isolates. For example, analyses of cellular respiration over 48 hours of incubation showed that ST313 isolates exploit particular carbon sources such as meso-tartaric acid (meso-tartrate) and tricarballylic acid more readily than *S*. Typhimurium ST19 isolates included in the experiment (Table 2). Conversely, the ST19 isolates, which includes SL1344, utilised carbon sources such as L-tartaric acid and dihydroxyacetone (Table 2). The differing metabolism of L-tartaric acid and meso-tartaric acid by ST313 and ST19 corroborates the observation that *ttdA*, encoding the stereo-specific enzyme tartrate dehydratase, is a pseudogene in ST313 isolates [27,28] [29].

**Table 2:** Summary of metabolites with significantly different impact on respiration.

Test metabolite / comparison	P-adjusted	Signal value difference
**48 hours: SL1344 vs. lineages 1 & II**		
Dihdroxy Acetone	3.24E-02	123.71
L-Tartaric Acid	1.49E-03	153.96
Tricarballylic Acid	6.10E-04	-198.57
M-Tartaric acid	4.19E-08	-262.43
**48 hours: lineage 1 vs lineage II**		
No significant results		
**15 hours: SL1344 vs. lineage I**		
Guanosine- 2'—monophosphate	1.90E-05	104.27
pH 4.5 + L-Histidine	8.00E-02	110.03
D-Glucosaminic acid	9.63E-03	113.88
Cytidine—2', 3'—cyclic monophosphate	3.98E-11	115.94
Guanosine- 3'—monophosphate	1.16E-05	115.97
pH 4.5 + L-Aspartic acid	4.07E-02	126.93
Cytidine—3'—monophosphate	6.65E-06	129.25
Uridine-3'-monophosphate	6.11E-06	132.29
Uridine- 2',3' cyclic monophosphate	1.37E-11	134.18
Cytidine-2'- monophosphate	1.90E-05	138.46
Tricarballylic acid	3.32E-02	-135.86
M-Tartaric acid	5.90E-02	-85.28
**15 hours: SL1344 vs. lineage II**		
Oxalomalic acid	8.02E-12	185.83
Cytidine- 2'—monophosphate	1.12E-07	148.82
Uridine-3-monophosphate	3.41E-07	136.92
Uridine-2-rsquo;3'-cyclic monophosphate	2.83E-12	136.14
Cytidine- 3'-monophosphate	1.92E-08	135.73
D-Glucosamic acid	8.18E-07	129.02
Guanosine-5'-monophosphate	9.46E-12	124.43
Cytidine-2-3'-cyclic monophosphate	2.83E-12	116.93
alpha-methyl-D-Galactoside	1.20E-05	116.19
Bromo Succinic acid	6.93E-04	115.53
Guanosine-3'—monophosphate	1.85E-06	108.37
M-tartaric acid	3.21E-03	-71.64
Tricarballylic acid	6.87E-03	-121.87
**I5 hours: lineages I & II vs. non-ST313**		
Uridine- 2-3'-cyclic monophosphate	5.15E-18	-140.74
Cytidine-3'-monophosphate	1.05E-14	-138.78
Cytidine-2'-monophosphate	1.06E-12	-134.05
D-Glucosaminic acid	7.35E-10	-132.12
Cytidine- 2-3'- cyclic monophosphate	7.63E-16	-124.80
Melibionic Acid	3.03E-03	-109.58
Guanosine- 3'-monophosphate	1.09E-08	-101.13

Negative signal value difference represent metabolites that were utilised more by isolates in lineages I and II; positive signal values represent metabolites utilized more by SL1344 or other non-ST313 isolates. Significance is reported at signal values ≥ 100 and *p*-adjusted values of ≤ 0.05. Results similar to the 48 hour profiles with adjusted *p*-values of <0.05 were also reported for the 15 hour profiles.

### *S*. Typhimurium ST313 isolates exhibit reduced enteropathogenicity

**Fig 4:**
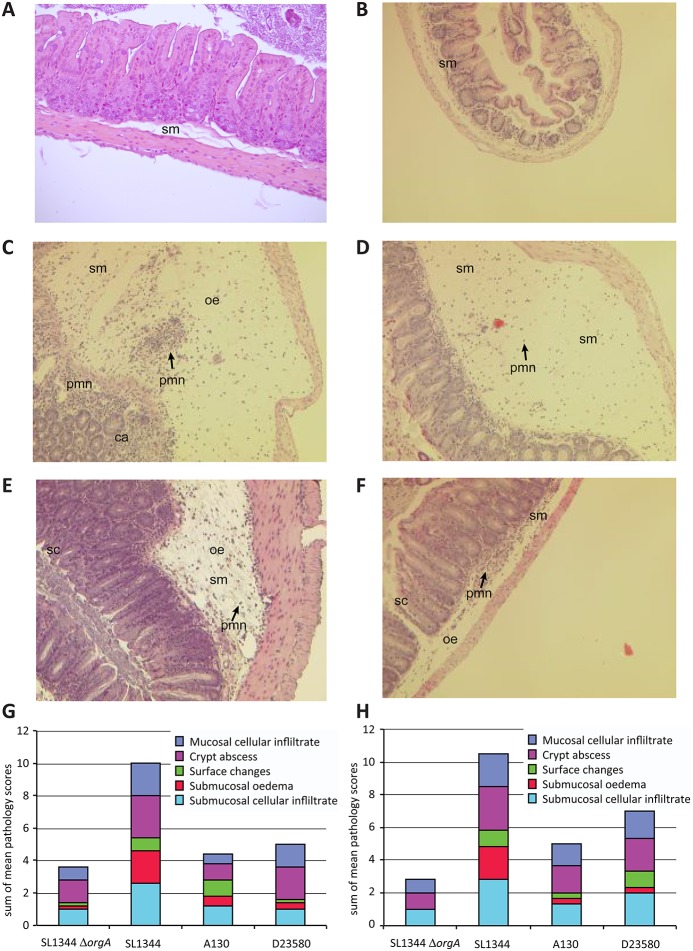
Histopathological analysis of *Salmonella* induced caecal inflammation of streptomycin-pre-treated mice. A single representative caecal wall image from each group is shown. **A**. naïve uninfected mouse. **B**. Infection with SL1344Δ*orgA* (SPI-1 mutant derivative). **C & D**. Infection with SL1344. **E**. Infection with A130 (lineage 1); **F**. Infection with D23580 (lineage II). Images A, C, D, E & F were taken at x100 magnification of original size (image B is x50 magnification). Abbreviations: l, intestinal lumen; sm, submucosa; oe, submucosal oedema; pmn (polymorphonuclear leukocytes) infiltrate; sc, surface changes; ca, crypt abcesses. Histopathology score of inflammatory changes in the caecum of streptomycin pretreated C57bl/6 mice (**G**) or 129P2/olaHsd mice (**H**) with ST313 isolates or strain SL1344, two or three days post inoculation. The scores of five inflammatory markers according to the key are indicated.

To determine if ST313 isolates are virulent in a mouse systemic infection model we orally inoculated genetically susceptible mice (NRAMP1^-^, C57bl/6) with representative ST313 isolates from lineage I and II. The resulting data showed that the tested ST313 isolates are indeed able to colonise systemic sites in this model (S3 Fig). We therefore investigated the ability of representative isolates of ST313 (A130 from lineage I and D23580 from lineage II) to induce an inflammatory response in the caecum of orally inoculated streptomycin pre-treated C57bl/6 mice, compared with SL1344 (Fig. 4). No significant difference in *Salmonella* colonisation of the caecum was evident at 48 hours post-inoculation (S4 Fig). SL1344 induced pronounced inflammation characterised by marked oedema in the submucosa with moderate to marked cellular inflammatory infiltrates in the submucosa and mucosa, with numerous crypt abscesses and erosive changes in the surface epithelium (Fig. 4C & 4D). However, these pathological signatures were less common in mice infected with A130 (Fig. 4E) or D23580 (Fig. 4F), although there was some evidence of mild to moderate submucosal oedema and mild inflammatory cell infiltration into the submucosa and mucosa. The epithelial surface changes and crypt abscesses were also much less prominent. SL1344Δ*orgA*, a SPI-1 defective derivative induced similar levels of inflammatory cell infiltration into the mucosa and submucosa of the caeca to A130 and D23580 (Fig. 4B). Uninfected caecum exhibited no noticeable oedema or neutrophil infiltration (Fig. 4A). The histopathological scores of the replicate experiments summarised in Fig. 4G & 4H illustrate these observations. In further experiments, groups of streptomycin pretreated 129P2/olaHsd mice were independently inoculated with the same *S*. Typhimurium isolates as in the previous experiment in C57bl/6 mice. Similar differences in intestinal pathology in the caecum were observed 48 hours post-inoculation (Fig. 4H).

**Fig 5:**
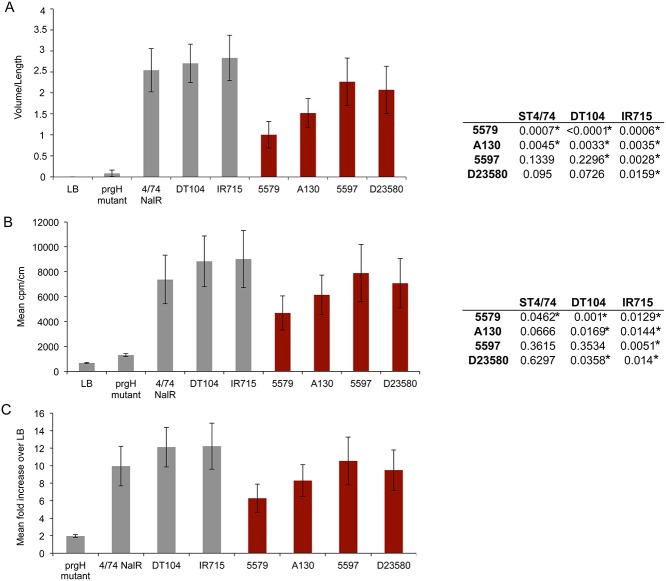
Secretory and inflammatory responses induced by ST19 (grey bars) and ST313 *S*. Typhimurium strains (maroon bars) in bovine ligated ileal loops. **A**. Mean fluid accumulation normalised to loop length [volume (mL)/length (cm)]. **B**. Influx of ^111^In-labelled PMN for test strains normalized to loop length and negative control loops, as described in Methods. Values represent the mean ± SEM of triplicate determinations in two independent calves. Right panel of A and B shows summary of paired T-test results. Asterisks show statistically significant pairwise comparisons. **C**. Influx of ^111^Indium oxinate-labelled polymorphonuclear leukocytes (PMN) induced by ST313 isolates relative to the negative control.

The overall virulence profiles observed in the streptomycin-treated mouse model of colitis were also evident in a bovine ligated ileal loop model. In these studies, ligated segments of the mid-ileum of two calves were infected in triplicate with representative invasive ST313 isolates from the two lineages (lineage I—A130 & 5597; lineage II- D23580 & 5579) and compared to bovine virulent ST19 *S*. Typhimurium strains ST4/74, DT104 and IR715 (S1 Table) and internal negative controls. Secretory and inflammatory responses in this model are strongly influenced by SPI-1 (*prgH* mutation; Fig. 5), as previously described [30]. In pair-wise *t*-tests, a significant difference in fluid accumulation was detected 12 hours post-inoculation between ST19 and ST313 isolates in almost all cases (Fig. 5). Mean values for the secretory response to the three ST19 isolates also differed significantly from the mean value for the four ST313 isolates (*p* = 0.02). Recruitment of ^111^Indium oxinate-labelled polymorphonuclear leukocytes (PMN) relative to the negative control (PMN influx) also differed significantly for a number of ST19 and ST313 isolates in pair-wise combinations (Fig. 5). Though the difference in mean values for PMN influx for all ST19 vs. ST313 was marginally not significant (*p* = 0.065), the difference was significant for PMN recruitment to the luminal contents by ST19 vs. ST313 (*p* = 0.04).

## Discussion

Here, we have identified lineage-specific signatures and phenotypic changes that differentiate ST313 from other *S*. Typhimurium, including isolates associated with gastroenteritis. These findings extend on previous analyses of D23580, a lineage II ST313 isolate to the broader ST313 population [5]. We identified and highlighted lineage specific gene acquisition and loss events, some common to both ST313 lineages and others restricted to either lineage I or II. Among the collective changes that have accompanied the emergence of ST313 are a relatively high proportion of genomic changes found in metabolic genes (Fig. 2A). This is worthy of note since altered metabolic capacity has previously been associated with adaptation of *Salmonella* serotypes to extra-intestinal niches [31]. The high numbers of SNPs within this class of gene could also be indicative of evolutionary pressure acting on the ST313 isolates. Examples of genes within this group include *ttdA*, that are also found either deleted or are pseudogenes in host-restricted or host-adapted serovars such as *S*. Typhi, *S*. Paratyphi A, *S*. Paratyphi B and *S*. Gallinarum. This ability to utilise less common carbon sources such as meso-tartrate and tricarballylic acid by the ST313 isolates may positively influence their fitness in a new ecological niche.

Degradation of aerobic metabolic genes in the isolates of the two lineages may suggest a preferential loss or reduction of aerobic metabolic capacity in ST313. This observation could be indicative of a heightened ability for anaerobic metabolism following internalisation within macrophages, as anaerobic respiration and metabolism takes precedence over aerobic metabolism within this niche. These metabolic activities impact the interaction of the pathogen with the host in the intracellular niche and have implications for intracellular compartmentalisation within tissues such as the bone marrow, as previously reported [32,33].

Further evidence for the clonality of the two epidemic lineages in sub-Saharan Africa is emphasized by the predominantly conserved pattern of the known *S*. Typhimurium phages and genomic islands. Although we present details of common SNPs in ncRNAs found in intergenic regions of the genomes in ST313 (S1 Text), the impact of these SNPs on regulation and subsequently on metabolism or virulence-associated phenotypes are difficult to predict and will be the subject of future investigation.

Surface proteins are often antigenic in nature and are part of the first line of contact with the host immune system. Changes in surface proteins can thus impact on the host response to colonisation and invasion by these pathovariants. The so-called ‘stealth’ methods employed by host-adapted serovars to evade host gut inflammatory responses leading to increased invasive capability have been well documented [34]. Thus, potential inactivation of genes such as *pagO* [23,25], *pipD* [22], *ratB* [20] and *sseI* [5] in ST313 isolates is interesting in this regard (Table 3). These proteins are all exported or membrane surface-associated proteins implicated in the establishment of gastrointestinal infection or long-term systemic infections in animal models. Such defects have been observed in the differential virulence profiles observed in pigs infected with *S*. Typhimurium, which cause non-fatal but acute enteritis, and *S*. Choleraesuis, which is host-adapted and frequently causes a severe systemic disease in pigs. In a porcine ligated ileal loop model, *S*. Typhmiurium elicits a profound inflammatory response, which subsequently controls and confines the pathogen to the intestinal mucosa. Conversely, the host-adapted *S*. Choleraesuis replicated slowly and elicited weaker pro-inflammatory responses both of which may facilitate avoidance of the host immune response by stealth [35]. Although we do not show a direct causal link, it is also possible that the inactivation of these virulence-associated proteins has led to the reduction in the enteropathogenic potential of isolates in the lineages I and II (Fig. 4 & 5, respectively).

**Table 3:** Gene accession numbers/IDs.

Gene	Accession number	
	Uniprot	EMBL
*allB*	E1W930	CBW16615
*gcl*	E1W925	CBW16610
*gip*	E1W926	CBW16611
*orgAa*	E1WAB5	CBW18948
*orgAb*	E1WAB4	CBW18947
*pagM*	E1WGA4	CBW17891
*pagO*	E1WGA0	CBW17887
*phnT*	E1W8T8	CBW16522
*phoP*	E1WFA1	CBW17265
*phoQ*	E1WFA0	CBW17264
*pipD*	E1W7C5	CBW17129
*prgH*	E1WAB9	CBW18952
*ratB*		CBW18577
SL3051	E1WHL5	CBW19150
SL1475	E1WBM7	CBW17570
SL1567	E1WBW8	CBW17662
SL1873	E1WGH9	CBW17967
SL2214	E1WC80	CBW18310
SL2653	E1WJG7	CBW18755
SL2659	E1WJH3	CBW18761
SL2990	E1WAQ5	CBW19089
SL3733	E1WDJ3	CBW19825
SL4223	E1WEX6	CBW20309
*sse1*	E1WCC7	CBW18358
*ttdA*	E1WID8	CBW19423
*ybbw*	E1W929	CBW16614
*ybjZ*	E1W6X5	CBW16977
*yhjU*	E1WD54	CBW19683

SL1344 gene names when not given are abbreviated to SLxxxx

*S*. Typhimurium ST313 is frequently associated with iNTS disease in sub-Saharan Africa. However, the extent to which this genotype is also associated with gastroenteritis in this region is poorly understood. iNTS disease syndrome is distinct from typhoid fever and gastroenteritis and thus lack an established animal model of infection. Mice with a defective *Nramp1* gene are also susceptible to invasive NTS disease, so we used this infection model to determine if ST313 isolates differed in their ability to colonise systemic sites organs of the reticuloendothelial system and the gall bladder compared with the non-ST313 SL1344. All ST313 exhibited some virulence in the mice and colonised to a similar level to that observed for SL1344 (S3 Fig). This is consistent with reports that ST313 isolates can establish systemic infections in different models of infection [36]. We also evaluated the ability of ST313 isolates (A130, D23580) and ST19 (SL1344) to invade eukaryotic cells growing *in vitro* was using Hep2 cells. Although, all three isolates showed evidence of invasion, internalisation and replication in epithelial cells over a time course of 24 hours, A130 and D23580 consistently showed lower invasion compared to SL1344 in this *in-vitro* model (*p* < 0.001 at 24 hours) (S5 Fig, SI Text).

We have detailed the shared genomic and phenotypic variation that may contribute to the adaptation of these new pathovariants to the novel niche provided by immunocompromised humans, identifying several changes that are consistent with those found in host-adapted lineages of *S*. *enterica*. The high proportion of metabolic genes implicated in the degraded gene component in lineages I and II of ST313 is a signature that is an emerging narrative among invasive pathogens in enterobacteriaceae including *Salmonella* [16], *Shigella* [37], *Yersinia* [38] and *E*. *coli* [39]. Our results thus suggest adaptation within a particular human population in ST313. However, the possibility of asymptomatic carriers or environmental reservoirs being integral components of iNTS transmission networks also exists. Elucidating these networks and defining the relationship between zoonotic, environmental and human isolates remains the subject of much needed on-going research.

## Methods

### Bacterial isolates and culture conditions

Bacterial isolates used in this study have been described in Okoro, *et al*., 2012[40]. See S1 Table. All bacteria were grown on Luria-Bertani (LB) medium; single colonies were incubated in LB Broth overnight at 37°C. Descriptions of specific growth conditions for experiments are given in the corresponding segments below.

### Calculating dN/dS of identified lineages

dN/dS was calculated using the formula adapted from Holt *et al*., 2008 (*N/n*)/(*S/s*), where *N* = sum of nonsynonymous SNPs, *n* = nonsynonymous sites in non-repetitive protein-coding sequences, *S* = sum of synonymous SNPs, s = synonymous sites in non-repetitive protein-coding sequences[14,41]

### Functional characterisation of SNP categories

To investigate the origin of SNPs reported on the tree, SNPs were reconstructed back to the phylogenetic tree using parsimony and optimised by both ACCTRAN (accelerated transformation) and DELTRAN (delayed transformation)[42]. Both methods gave comparable results and so the results from the DELTRAN optimisation are presented here. The DELTRAN method allocates or maps SNP origins along the phylogenetic branches as close to the tips as possible [43]. This enabled frameshift mutations and premature stop codons that reduced the length of CDSs relative to their annotation in the reference genome to be detected. SNP positions, type and quality were manually confirmed by checking reads against the reference sequence and visualised using BamView[44].

### Detection of distribution of insertion sequences, phages and pathogenicity islands by mapping

Paired-end sequence reads of each isolates were mapped to the multi-fasta sequence features of either insertion sequences, phages or pathogenicity islands using the Burrows-Wheeler Aligner software BWA[45], with minimum base call quality of 50, minimum mapping quality of 30, and minimum read depth of 4. Isolates from each of identified lineages were analyzed separately, by lineage. A cut-off value of < 30% of reads mapped to the length of the feature was selected as an indication of absence and > 70% as presence of the region of interest in an isolate. A heat map of the analysis based on the selected cut-off values was generated.

### Biolog experiments

Culture and inoculum preparation were preformed according to a modified manufactures’ protocol (see S1 Text). A total of 576 assays were performed for each isolate, with each isolate represented by three biological replicates. Bacteria were incubated for 15–48 hours at 37°C and bacterial respiration on each assayed metabolite was measured by colorimetric redox assay. The metabolic activity and kinetics data files of each strain over time were exported from the OmniLog phenotype MicroArray (PM) program suite. Further analysis proceeded as described previously in R[46]. Signal values were calculated as in Homann *et al*., 2005 [47]. Log signal values displayed a clear bimodal distribution corresponding to non-respiring (background dye reduction) and respiring modes. Normal distributions were fitted to each mode, and strains were defined as respiring on a particular substrate if all 3 replicates were at least 4 times more likely to originate from the respiring distribution. Significant differences in respiration rates between isolates were assessed using a moderated t-test with the LIMMA R package [48]. *P*-values were corrected using the Benjamini and Hochberg method [49] to control for the false discovery rate. Results presented here are for respiration for up to 15 hours and 48 hours. Results with adjusted *p*-values of <0.05 and signal value differences (positive or negative) greater than or equal to 100 at 48 hours were selected as significant. Results similar to the 48 hour profiles with adjusted *p*-values of <0.05 were also reported for the 15 hour profiles.

### Determination of common phenotypes and metabolic pathway analyses

The functions of metabolites significantly utilized to a greater or lesser degree by the invasive isolates commonly relative to SL1344 were identified for each cluster, and the list of the associated metabolites generated and analysed with Pathway Tools [50] to put them in a wider context and predict the metabolic pathways that were involved.

### Ethics statement

All mouse experiments were conducted in compliance with the Animals (Scientific Procedures) Act 1986 under Home Office project licence 80/2596 with the consent and approval of the Ethical Review Committee of the Wellcome Trust Sanger Institute, UK. Mice were sacrificed by cervical dislocation at the end of the experiment. Calf ligated ileal loop experiments were conducted in compliance with the Animals (Scientific Procedures) Act 1986 under Home Office project licence 30/2485 with the consent and approval of the Ethical Review Committee of the Institute for Animal Health, UK. General anaesthesia was induced by intravenous administration of propofol and maintained by inhalation of isoflourane in oxygen for the duration of the study. Calves were given an overdose of intravenous sodium pentobarbitone at the end of the study.

### Streptomycin pre-treated mouse model of colitis

*S*. Typhimurium isolates were grown in LB agar supplemented with appropriate antibiotic selection and incubated overnight at 37°C. Single colonies were used to inoculate LB broth and incubated overnight at 37°C. Approximately 1x10^7^ were inoculated into each mouse. Two experiments were conducted and a total of 5 and 3 mice per isolate were used for the infections in first and second experiment, respectively. Specific pathogen-free SPF female mice C57BL/6 (groups of five) or female 129P2/olaHsd mice, (groups of three), 6–8 weeks old, were treated by oral gavage with 0.2 mL of 100 mg/mL streptomycin by oral gavage. At 20 hours after streptomycin treatment, mice were infected with 1x10^6^ (C57BL/6 mice) or 1x10^7^ (129P2/olaHsd mice) of *S*. Typhimurium in 0.2 ml of PBS pH 7.4 or treated with sterile PBS (control) by oral gavage. At 48 hours (C57BL/6) and 72 hours (129P2/olaHsd), mice were culled and two caecal tissue samples taken for enumeration of viable *S*. Typhimurium or were fixed with formalin for subsequent wax embedding, sectioning and tissue staining with haematoxylin/eosin (H/E) staining. Enumeration of bacteria was conducted by plating serial dilutions of caecal tissue homogenates on LB agar containing the appropriate antibiotics. Colonies were counted after overnight incubation at 37°C. The H/E stained caeca were histopathologically assessed and scored using a 4-point scale of 0, 1, 2, or 3, for five markers of vascular and cellular inflammation by using a modification of methods described in Kim, J.J. *et al*., 2012 [51] as follows; mucosal inflammatory cellular infiltration predominantly by neutrophils (PMNs), the presence of crypt abscesses (neutrophils within the lumen of the crypts in the mucosa), erosive and reactive changes to the epithelial surface of the mucosa, the amount of submucosal oedema assessed by the increase in thickness of the submucosa and the level of submucosal inflammatory cellular infiltration predominantly by neutrophils.

### Bovine ligated ileal loop experiments

*Salmonella*-induced secretory and inflammatory responses in calves were quantified essentially as described previously [30]. Briefly, two 4-week-old Friesian bull calves were placed under terminal general anaesthesia, a laparotomy performed and the mid-ileum flushed with sterile PBS. In each calf, twenty-seven 6 cm loops with 1 cm spacers were constructed by ligation of the gut with surgical silk. Representative invasive ST313 isolates from the two lineages (lineage I—A130 & 5597; lineage II- D23580 &5579) and bovine virulent ST19 strains SL1344, DT104 and IR715. IR715 is a nalidixic acid-resistant derivative of strain ATCC 14028 [52]. Triplicate loops in each calf were inoculated in a semi-randomized order with c. 1x10^9^ CFU of the indicated *S*. Typhimurium strains grown to mid-logarithmic phase in LB broth at 37°C. Three loops in each calf were inoculated with an equivalent volume of sterile LB broth as a negative control. After inoculation the mid-ileum was returned to the abdominal cavity for 12 h then the animals given an overdose of pentobarbitone sodium. At *post mortem* examination, loops were excised and the volume of fluid accumulated recorded and normalized to loop length [volume (mL)/length (cm)]. To quantify inflammation, c. 80 mL of jugular blood was collected at the start of the experiment and PMN isolated and labelled with ^111^Indium oxinate as described[30] then injected into the donor calf within 1 h of loop inoculation. Gamma-radioactivity associated with the mucosa and contents of each loop was normalized to loop length (counts per minute/cm), then the mean PMN influx for each set of triplicate loops determined by dividing the mean value for test strains by the value for the negative control. Values shown are the mean ± standard error of the mean (SEM) from two independent animals.

## Supporting Information

S1 Text Supporting information text, methods and references. (DOC)

S1 Fig Relationship between *S*. Typhimurium (including the two ST313 lineages) and selected *S*. *enterica* serovars. Maximum likelihood tree inferred from concatenated SNPS in the core genes obtained using the Panseq program[53]. Scale bar indicates substitutions per variable site. Nodes with 100% bootstrap support are indicated by asterisks.
(TIF)

S2 Fig Heatmap of log signal values from phenotypic tests. The data illustrates the extent of variability within replicates and the clustering of isolates based on overall metabolic potential on tested metabolites and physiological conditions.
(TIF)

S3 Fig Numbers of (cfu) recovered from liver, bone marrow and gall bladder of *S*. Typhimurium susceptible C57bl/6J mice. Geometric mean of recovered bacteria (cfu/ml) from mice infected with ST313 isolates (x-axis). Dotted lines running parallel to x-axis indicate numbers (geometric mean) recovered bacteria from SL1344 infections (grey vertical lines).
(TIF)

S4 Fig Caecal bacterial loads in streptomycin pre-treated mice (*p* < 0.05; Mann-Whitney, two tailed test for significance). (TIF)

S5 Fig Levels of ST313 isolates A130 (blue bars) and D23580 (red bars) and SL1344 (grey bars) expressed as a % of recovered cells from Hep2 to initial inoculum. Asterisks indicate statistically significant differences at *p* < 0.001 using a two-way ANOVA with post-tests performed using the Bonferroni method.
(TIF)

S1 Table Isolates used in study. * ST4/74 is the parent of SL1344 and differs from it by just 8 SNPs. ST4/74 is the strain used Biolog and bovine experiments. For consistency, ST4/74 is referred to as SL1344 in the relevant sections within the text. **IR715 is a nalidixic acid-resistant derivative of strain ATCC 14028.
(XLS)

S2 Table Deleted loci in ST313 *S*. Typhimurium isolates. Boundaries of deleted loci are indicated in the first two columns. SL1344 gene names when not given are abbreviated to SLxxxx.
(XLS)

S3 Table Pseudogenes in ST313 *S*. Typhimurium lineages. Single asterisks indicate pseudogenes common to all isolates within a particular lineage. Lineages without asterisks indicate branches leading to terminal nodes. Gene categories were identified using Artemis-based gene classification scheme. SL1344 gene names when not given are abbreviated to SLxxxx.
(XLS)
