# Correction: Signatures of Adaptation in Human Invasive *Salmonella* Typhimurium ST313 Populations from Sub-Saharan Africa

**DOI:** 10.1371/journal.pntd.0003848

**Published:** 2015-06-15

**Authors:** Chinyere K. Okoro, Lars Barquist, Thomas R. Connor, Simon R. Harris, Simon Clare, Mark P. Stevens, Mark J. Arends, Christine Hale, Leanne Kane, Derek J. Pickard, Jennifer Hill, Katherine Harcourt, Julian Parkhill, Gordon Dougan, Robert A. Kingsley

There are several errors in this published article.

In the penultimate sentence of part A the Fig 1 legend, the word “pseudogenes” in the parentheses should read “prgH”.

The table legend for Table 3 is missing. Please see the complete, correct Table 3 legend here.

**Table 3. Gene accession numbers/IDs**. The common gene name, uniprot ID and Embl accession for all genes described in the text are provided. SL1344 gene names when not given are abbreviated to SLxxxx.

In the fourth sentence of the fourth paragraph of the Discussion, Table 3 is incorrectly linked to. This sentence should link to Fig 1. Please see the corrected sentence here.

Thus potential inactivation of genes such as pagO, pipD, ratB and sseI in ST313 isolates is interesting in this regard (Figure 1).
