# Correction: Correction: Signatures of Adaptation in Human Invasive *Salmonella* Typhimurium ST313 Populations from Sub-Saharan Africa

**DOI:** 10.1371/journal.pntd.0003970

**Published:** 2015-08-07

**Authors:** The PLOS Neglected Tropical Diseases Staff

This correction is being issued to modify a previous correction. The second sentence should be modified to the following: Figure 1. Due to a typographical error, prgH is mistakenly listed as a pseudogene.

## References

[1] Okoro CK, Barquist L, Connor TR, Harris SR, Clare S, Stevens MP, et al (2015) Correction: Signatures of Adaptation in Human Invasive *Salmonella* Typhimurium ST313 Populations from Sub-Saharan Africa. PLoS Negl Trop Dis 9(6): e0003848 doi:10.1371/journal.pntd.0003848 26076129 10.1371/journal.pntd.0003848 PMC4468169

