# Supplementary material for: Signatures of Adaptation in Human Invasive Salmonella Typhimurium ST313 Populations from Sub-Saharan Africa
Source: PLoS Negl Trop Dis. 2015 Mar 24;9(3):e0003611. doi: 10.1371/journal.pntd.0003611 (PMC4372345; doi:10.1371/journal.pntd.0003611)
Supplement: S1 Text — (DOC) [file pntd.0003611.s001.doc]

**Signatures of adaptation in human invasive *Salmonella* Typhimurium ST313 populations from sub-Saharan Africa**

Chinyere K. Okoro*^1^, Lars Barquist^1,2^, Thomas R. Connor^1,3^, Simon R. Harris^1^, Simon Clare^1^, Mark P. Stevens^4^, Mark J. Arends^5,6^, Christine Hale^1^, Leanne Kane^1^, Derek Pickard^1^, Jennifer Hill^1^, Katherine Harcourt^1^, Julian Parkhill^1^, Gordon Dougan^1^ Robert A. Kingsley^1,7^

^1^Wellcome Trust Sanger Institute, Wellcome Trust Genome Campus, Hinxton Cambridge, UK. CB10 1SA

^2^Institute for Molecular Infection Biology, University of Wuerzburg. Josef-Schneider-Strasse 2 / Building D15. D-97080 Wuerzburg, Germany

^3^Cardiff School of Biosciences, Cardiff University. Sir Martin Evans Building. Museum Avenue, Cardiff , UK. CF10 3AX

^4^The Roslin Institute & Royal (Dick) School of Veterinary Studies. University of Edinburgh. Easter Bush, Midlothian, Scotland, UK. EH25 9RG

^5^Department of Pathology, University of Cambridge, Addenbrokes Hospital, Hills Road, Cambridge, UK. CB2 2QQ

^6^Edinburgh Cancer Research Centre, University of Edinburgh, Western General Hospital, Crewe Road South, UK. EH4 2XR

^7^Institute of Food Research, Norwich Research Park, Colney Lane, Norwich, Norfolk NR4 7UA

*Corresponding author

**Supporting Information**

**Characterising intergenic SNPS in ST313 lineages**

We investigated the proportions of homoplasies in the SNP repertoire of the two ST313 lineages. The proportions of homoplasic SNPs were uniformly low within ST313. Exceptions were observed with the homoplasies found on the branch labelled ‘50’ (Fig. 1A), which leads to lineages I and II and branch ‘51’ leading to lineage I. In both cases, homoplasies made up >10% of total SNPs (Table 1). Most of the homoplasic SNPs were found within intergenic regions of the chromosome. Because it is possible that these intergenic SNPs may fall in regulatory regions , and thus be under selection, we probed the sequences of isolates in lineages I and II for conserved SNPs in non-coding RNA (ncRNA) drawn from the Rfam database and a previous study of ncRNA function in salmonellae . In lineage I, we found two SNPs in tmRNA, an RNA with partial homology to tRNAs. tmRNA contains a short ORF encoding a protein degradation signal responsible for freeing stalled ribosomes. It has also been shown to be important for survival in stress conditions, including proliferation in macrophage . In addition to these, a single SNP was found in one copy of StyR-44 , a multi-copy sRNA associated with ribosomal operons previously suggested to play a role in the maturation of the 16S and 23S ribosomal subunits . Isolates in both lineages had a single SNP each in two uncharacterized sRNAs STnc1220 and STnc280 encoded within pathogenicity islands . The former is antisense to the *ssaK*, a *Salmonella* pathogenicity island 2 (SPI-2)-associated gene . However, unlike in *S.* Typhi or *C. difficile* the identified homplasic SNPs were not associated with chromosomally-borne antibiotic resistance genes.

**Candidate large deletions present in some ST313 isolates**

We identified deletions in the draft genome sequence of ST313 isolates was by mapping the raw reads to a reference genome assembly. This method has limitations for identification of large deletions. Within the constraints of the insert size of the mapped reads (>200bp to >300bp) and the mapping algorithm used, three candidate large deletions (>20bp) were found in two gene clusters in ST313 isolates. One of these encodes genes *oadG2*, *oadA* and *oadB2* (2.4kb) involved in citrate fermentation (S2 Table). Another spanned the genes *ccmA,B,C,D,E,,F,G,H* (4.85-5.25 kb) encoding the system I cytochrome C complex . We found that the corresponding genomic regions of the strain D23580 genome assembly do not contain these deletions.

**Metabolic profiling of ST313 lineages**

A principal component analysis (PCA) and a hierarchical clustering of Biolog signal values from all culture conditions used in the analyses were used to query the global metabolic profiles of the selected isolates (Fig 3A and 3B). These analyses reveal two major clusters, the first made up of isolates from of ST313 lineages I and II and a second encompassing isolates outside of these two lineages. Both analyses highlight congruency to phylogenetic positioning when considering overall metabolic capacity of lineage I and II and supports the conclusion that ST313 isolates within lineage I and II share similar metabolic capacity that is distinct from other *S*. Typhimurium isolates that we tested. The PCA analyses also revealed a third population, which contained two replicates from 5581(lineage I) and three replicates of 5634 (ST19 *S*. Typhimurium). Both 5581 and 5634 have a close temporal and geographical association, as they were isolated from Kenya in 2003. A single replicate of PVI is found outside of the identified clusters. However additional analyses show that the variability between replicates of the same strain is confined within the same cluster and supports the conclusion that isolates within each cluster share similar metabolic capabilities. (S2 Figure).

Although the comparisons reported for non-ST313 lineages were taken from 15-hour incubation data, the omnilog signals were informative enough to register differences in respiration. These data show that there were no commonly utilised metabolites when lineages I, II and ST19 isolates were considered as a single group and compared to SL1344 (ST4/74), based on the criteria we used to test for significance. This confirms that although few genetic signatures are common in all lineages I and II isolates, they are more similar to each other metabolically than to other isolates that fall outside of both lineages. Results from incubation after 15 hours further indicated that the ST19 isolates utilized purine and pyrimidine nucleotides as sources of phosphorus more readily than the isolates from lineages I and II (Table 2). Cyclic and non-cyclic nucleosides and nucleotides are components of nucleic acids and are important for cell division, proliferation and growth. The potential impact of metabolic potential on replication of ST313 within the host or in the environment is still unknown.

**Supplementary Methods**

**Phylogeny of *Salmonella enterica* serovars**

Selected *Salmonella enterica* (chromosome) genome sequences were downloaded from the relevant sequence databases (S1 Table). Analyses of core genes and alignment of core genome SNPs were performed using the Panseq programme suite v2.0. The maximum likelihood phylogenetic tree was inferred from an alignment of concatenated core genome SNPs as previously described .

**Detection of deletions**

Multiplex libraries with a 200bp insert size were prepared using 12 unique index-tags, and sequenced to generate 54 or 76 base-pair (bp) paired-end reads using the Illumina Genome Analyser II System according to manufacturers’ protocol. Paired-end sequences of isolates were mapped to the reference (SL1344) sequence using both SSAHA2 and Burrows-Wheeler Aligner software (BWA) to compare results and deletions were detected by manually inspecting the coverage of sequence reads of isolates using Artemis and BamView. To confirm deletions, presence or absence of the region of interest was examined by blastn search.

**Modified Biolog Phenotypic Microarray Protocol for *Salmonella* (adapted from**

**1. Inoculum preparation**

Bacteria were grown on LB plates incubated for 24 hours at 37^o^C; three single colony from the LB plates was used to inoculate 10 mL of low salt LB medium to make three replicates and incubated overnight at 37 ^o^C. Two mL of culture was used to inoculate 18 mL low salt LB broth and further incubated until OD_600_ 0.5 - 0.6. Care was taken to keep the OD of replicates as consistent as possible. The culture were then pelleted by centrifugation at 4,600 rpm at RT for 10 minutes, resuspended in 2 mL PBS, washed and pelleted (x3) as previously described. Bacterial cells were then resuspended in 5 mL of PM inoculating fluid IF-0a and used for subsequent PM inoculation steps. All PM panels and reagents were brought to room temperature (30 minutes) before starting experiment.

**2. Preparation of PM inoculating fluids; IF-0 and IF-10a**

IF-0 was prepared by adding 25 mL of sterile water into bottle containing 1.2x IF-0. We pipetted 16 mL of this IF-0 into a 20 x 150 mm sterile capped test tube to prepare for cell suspension. The IF-0+dye inoculating fluid was prepared by adding 1.8 mL of dye mix and 23.2 mL of sterile water into bottle containing 125 mL of 1.2x IF-0. 50 mL of this IF-0+dye was the pipetted into a sterile glass bottle for inoculating PM 1- plates. The inoculating fluid IF-10a was prepared by adding 1.5 mL of dye mix and 23.5ml of sterile water into the bottle containing 125 mL of 1.2x IF-10. Finally, 25 mL of IF-10a + dye was pipetted into 120 mL sterile glass bottles and subsequently used for inoculating PM 10 plates.

**3. Inoculation of PM Panels/Plates**

**a. Steps 1 -2: Prepare Cell suspensions**

Two to 5 ML of cell suspension from step 1 above was transferred aseptically into sterile capped tube containing 16 mL IF-0 and stirred to ensure a uniform suspension and to achieve 42%±2T (transmittance) in the Biolog turbidometer. 10 mL of 42±2%T cell suspension was added to the sterile bottle containing 50 mL of IF-0+dye (1:5 dilutions). The resultant suspension was mixed gently to achieve a final density of about 85%T.

**b. Steps 3 – 5: Inoculate PM 10**

125 µL of the 85%T cell suspension from step 3a is transferred into the sterile, prefilled glass bottle containing 25 mL of IF-10+dye (1:200 dilution), mixed completely but gently, transferred into sterile reservoir, and used to inoculate PM 10 plates, 100 µL/well.

**c. Steps 6 – 7: Inoculate PM 1- 2**

22 mL of the 85%T cell suspension prepared from step 3a was then transferred into a new sterile reservoir and used to inoculate PM 1 and 2 plates, 100 µL/well.

**d. Steps 8 – 10: Inoculate PM 3 - 4**

280 µL of carbon source (sodium succinate 2 M/ferric citrate 200 µM, final concentration) was added to the remaining 28 mL of the 85% T cell suspension prepared in step 3a, mixed as described above and then transferred to a new sterile reservoir. The resultant suspension was used to inoculate PM 3 – 4 plates, 100 µL/ well.

**HEp-2 cell invasion assay**

HEp-2 cell invasion assays were performed using a modification of the gentamicin protection assay previously described in Isberg & Falkow, 1985 . Stock flasks of HEp-2 cells were harvested and the cell count assessed by mixing cells 1:1 with Trypan blue vital stain. The cells were diluted to 2x10^5^ viable cells/mL in supplemented Dulbecco’s modified Eagle’s medium (DMEM), and 0.5 mL volumes dispensed into each well of 24-well plates (Costar C3424). The plates were placed into a humidified CO_2_ incubator at 37^o^C and left for two days. Bacterial cells used for infecting the HEp-2 cells were first grown to mid-log phase in LB at 37^o^C and then aliquots used to inoculate fresh LB and incubated overnight without shaking at 37^o^C. On the day of the infection, each HEp2-cell containing well was aspirated and washed with three volumes of DMEM containing 10% HI FCS and 2 mM L-Glutamine and subsequently, wells were replenished with 0.5 mL medium containing 10% FCS and 2mM L-Glutamine. Aliquots of 50 μL diluted overnight cultures of bacteria (approximately 5x10^7^ cells) were utilised to infect each well of cells. This resulted in a multiplicity of infection (MOI) of 50 bacteria per HEp-2 cell. The plates were centrifuged at 1500 rpm for 5 minutes at 11^o^C to promote adherence and invasion of bacteria. The infection was allowed to proceed for 30 minutes at 37^o^C before the wells were washed twice with phosphate buffered saline (PBS) and the wells replenished with 0.5 mL medium supplemented with 100 μg/mL gentamicin. This was taken as time 0 in all cases. At 2, 5 and 24 hours, wells were washed twice with PBS and the HEp-2 cells disrupted by addition of 0.5 mL of 1% Triton (in PBS), and then incubated at room temperature for 5 minutes. Serial dilutions of disrupted cell mixtures as well as of the inoculum were plated on the appropriate selective plates and incubated overnight at 37^o^C. Control wells without bacteria were included in the experiment. Viable bacterial cell counts were taken and results were reported as the percentage of the initial inoculum that was intracellular.

**Systemic infection experiments in the murine model**

Six ST313 isolates from invasive lineage I - A16083, A24924, A130, A4283, C2167 and A13212; and three from invasive lineage II - A32796, A38589 and A39155, were used in inoculating 33 *Salmonella*-susceptible female NRAMP1^-^ C57bl/6 mice. 200 µL (1 in 10 dilutions in PBS, OD_600_ > 0.8) of overnight cultures of isolates were used for inoculation by oral gavage. Each strain was used to infect three mice. SL1344 was also included as the control. Mice were sacrificed on day 4 and bacteria were enumerated from liver, bone marrow and gall-bladder as previously described. All mice were 6 -7 weeks old at the beginning of experiments

**Supplementary References**

1. Vogel J (2009) A rough guide to the non-coding RNA world of Salmonella. Mol Microbiol 71: 1-11.

2. Burge SW, Daub J, Eberhardt R, Tate J, Barquist L, et al. (2013) Rfam 11.0: 10 years of RNA families. Nucleic Acids Res 41: D226-232.

3. Barquist L, Langridge GC, Turner DJ, Phan MD, Turner AK, et al. (2013) A comparison of dense transposon insertion libraries in the Salmonella serovars Typhi and Typhimurium. Nucleic Acids Res 41: 4549-4564.

4. Hebrard M, Kroger C, Srikumar S, Colgan A, Handler K, et al. (2012) sRNAs and the virulence of Salmonella enterica serovar Typhimurium. RNA Biol 9: 437-445.

5. Chinni SV, Raabe CA, Zakaria R, Randau G, Hoe CH, et al. (2010) Experimental identification and characterization of 97 novel npcRNA candidates in Salmonella enterica serovar Typhi. Nucleic Acids Res 38: 5893-5908.

6. Yu J, Schneiders T (2012) Tigecycline challenge triggers sRNA production in Salmonella enterica serovar Typhimurium. BMC Microbiol 12: 195.

7. Pfeiffer V, Sittka A, Tomer R, Tedin K, Brinkmann V, et al. (2007) A small non-coding RNA of the invasion gene island (SPI-1) represses outer membrane protein synthesis from the Salmonella core genome. Mol Microbiol 66: 1174-1191.

8. Kroger C, Dillon SC, Cameron AD, Papenfort K, Sivasankaran SK, et al. (2012) The transcriptional landscape and small RNAs of Salmonella enterica serovar Typhimurium. Proceedings of the National Academy of Sciences of the United States of America 109: E1277-1286.

9. Holt KE, Parkhill J, Mazzoni CJ, Roumagnac P, Weill FX, et al. (2008) High-throughput sequencing provides insights into genome variation and evolution in Salmonella Typhi. Nature genetics 40: 987-993.

10. He M, Sebaihia M, Lawley TD, Stabler RA, Dawson LF, et al. (2010) Evolutionary dynamics of Clostridium difficile over short and long time scales. Proceedings of the National Academy of Sciences of the United States of America 107: 7527-7532.

11. Richardson DJ (2000) Bacterial respiration: a flexible process for a changing environment. Microbiology 146 ( Pt 3): 551-571.

12. Kranz RG, Richard-Fogal C, Taylor JS, Frawley ER (2009) Cytochrome c biogenesis: mechanisms for covalent modifications and trafficking of heme and for heme-iron redox control. Microbiology and molecular biology reviews : MMBR 73: 510-528, Table of Contents.

13. Laing C, Buchanan C, Taboada EN, Zhang Y, Kropinski A, et al. (2010) Pan-genome sequence analysis using Panseq: an online tool for the rapid analysis of core and accessory genomic regions. BMC Bioinformatics 11: 461.

14. Laing C, Villegas A, Taboada EN, Kropinski A, Thomas JE, et al. (2011) Identification of Salmonella enterica species- and subgroup-specific genomic regions using Panseq 2.0. Infect Genet Evol 11: 2151-2161.

15. Okoro CK, Kingsley RA, Connor TR, Harris SR, Parry CM, et al. (2012) Intracontinental spread of human invasive Salmonella Typhimurium pathovariants in sub-Saharan Africa. Nat Genet 44: 1215-1221.

16. Bentley DR, Balasubramanian S, Swerdlow HP, Smith GP, Milton J, et al. (2008) Accurate whole human genome sequencing using reversible terminator chemistry. Nature 456: 53-59.

17. Harris SR, Feil EJ, Holden MT, Quail MA, Nickerson EK, et al. (2010) Evolution of MRSA during hospital transmission and intercontinental spread. Science (New York, NY) 327: 469.

18. Ning Z, Cox AJ, Mullikin JC (2001) SSAHA: a fast search method for large DNA databases. Genome research 11: 1725-1729.

19. Li H, Durbin R (2009) Fast and accurate short read alignment with Burrows-Wheeler transform. Bioinformatics 25: 1754-1760.

20. Carver T, Harris SR, Berriman M, Parkhill J, McQuillan JA (2012) Artemis: an integrated platform for visualization and analysis of high-throughput sequence-based experimental data. Bioinformatics 28: 464-469.

21. Carver T, Harris SR, Otto TD, Berriman M, Parkhill J, et al. (2012) BamView: visualizing and interpretation of next-generation sequencing read alignments. Briefings in bioinformatics.

22. Isberg RR, Falkow S (1985) A single genetic locus encoded by Yersinia pseudotuberculosis permits invasion of cultured animal cells by Escherichia coli K-12. Nature 317: 262-264.

23. Kingsley RA, Humphries AD, Weening EH, De Zoete MR, Winter S, et al. (2003) Molecular and phenotypic analysis of the CS54 island of Salmonella enterica serotype typhimurium: identification of intestinal colonization and persistence determinants. Infection and immunity 71: 629.
