# Supplementary figures and images for: Signatures of Adaptation in Human Invasive Salmonella Typhimurium ST313 Populations from Sub-Saharan Africa
Source: PLoS Negl Trop Dis. 2015 Mar 24;9(3):e0003611. doi: 10.1371/journal.pntd.0003611 (PMC4372345; doi:10.1371/journal.pntd.0003611)

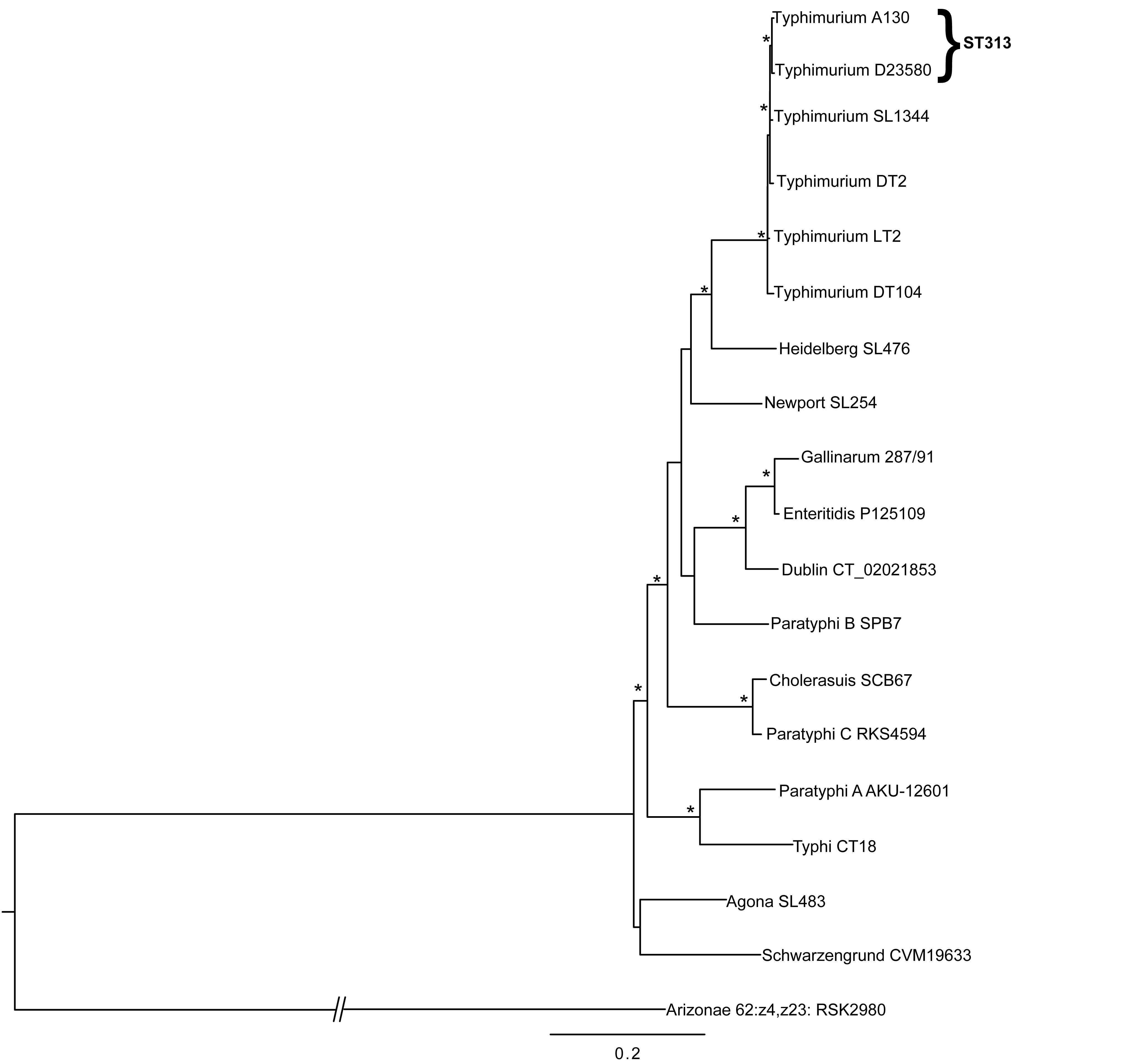

Supplement: S1 Fig — Maximum likelihood tree inferred from concatenated SNPS in the core genes obtained using the Panseq program[53]. Scale bar indicates substitutions per variable site. Nodes with 100% bootstrap support are indicated by asterisks. (TIF) [file pntd.0003611.s002.tif]

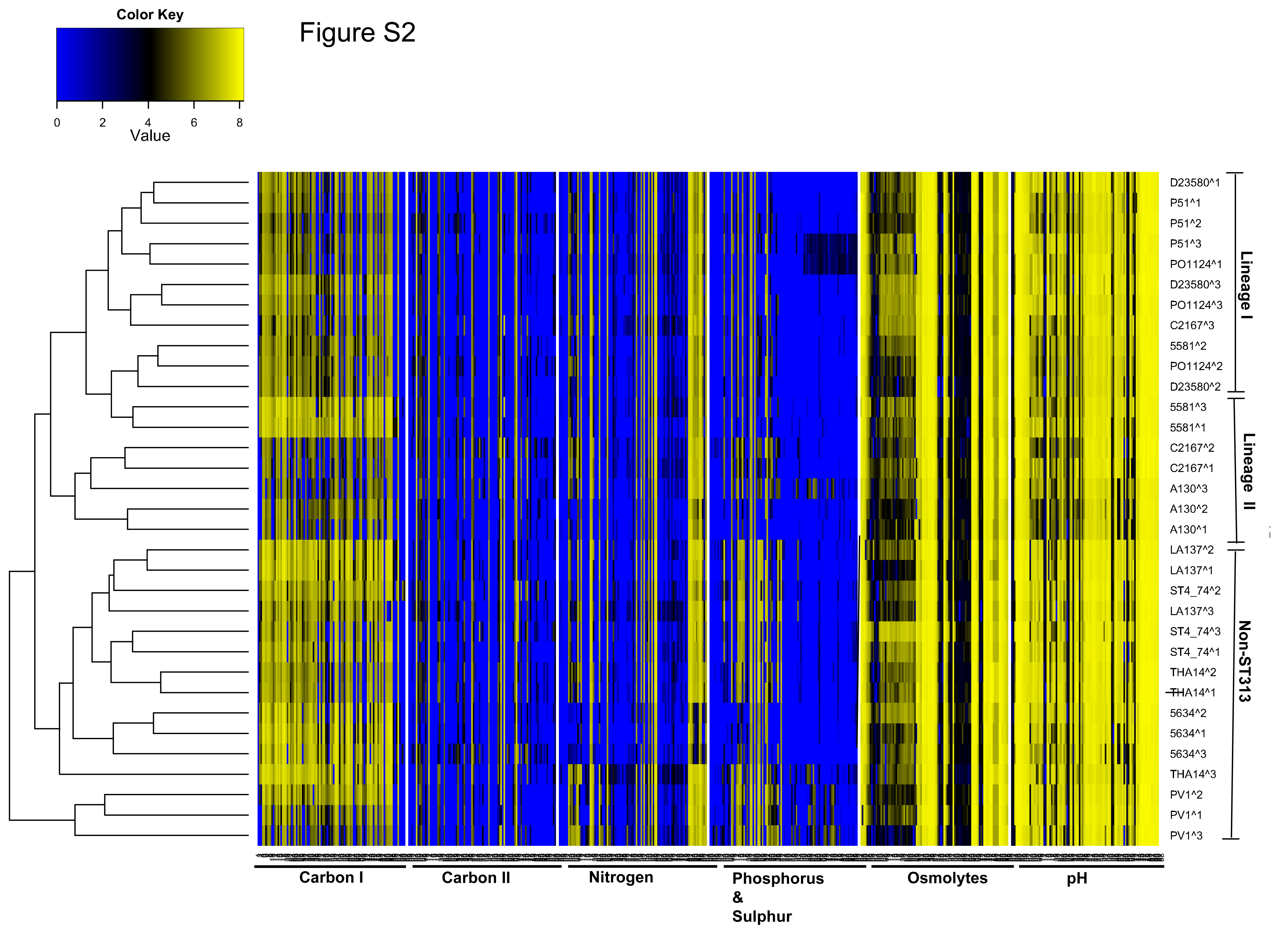

Supplement: S2 Fig — The data illustrates the extent of variability within replicates and the clustering of isolates based on overall metabolic potential on tested metabolites and physiological conditions. (TIF) [file pntd.0003611.s003.tif]

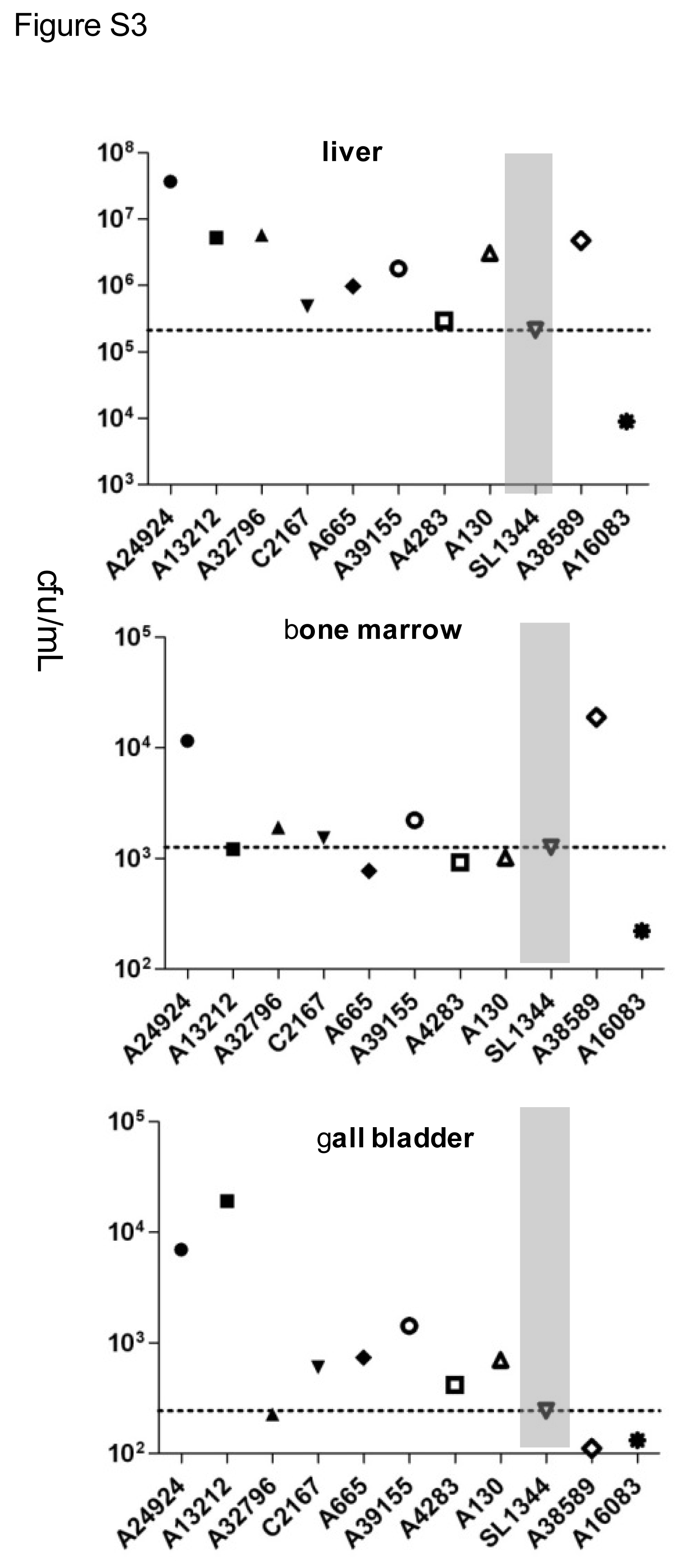

Supplement: S3 Fig — Geometric mean of recovered bacteria (cfu/ml) from mice infected with ST313 isolates (x-axis). Dotted lines running parallel to x-axis indicate numbers (geometric mean) recovered bacteria from SL1344 infections (grey vertical lines). (TIF) [file pntd.0003611.s004.tif]

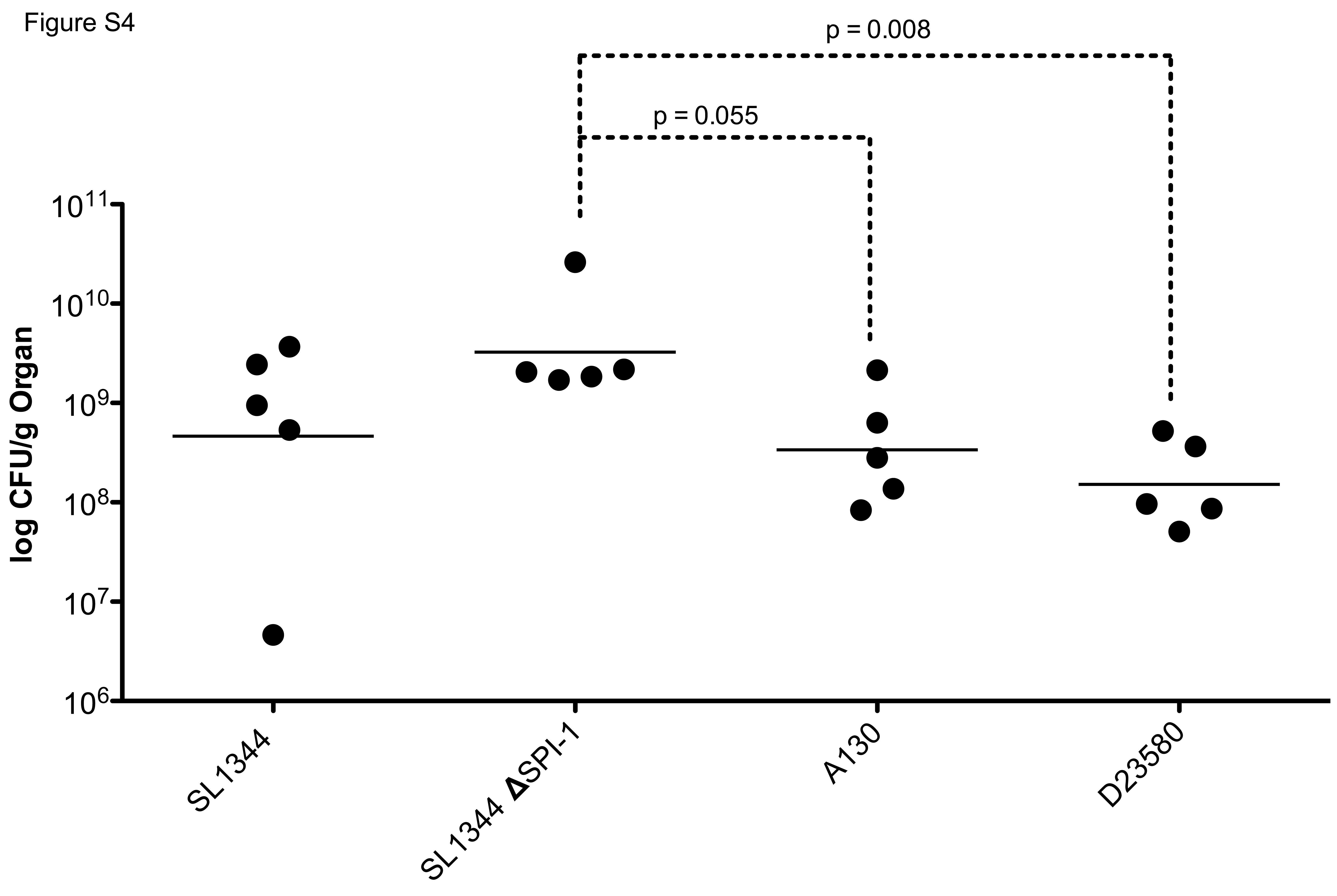

Supplement: S4 Fig — (TIF) [file pntd.0003611.s005.tif]

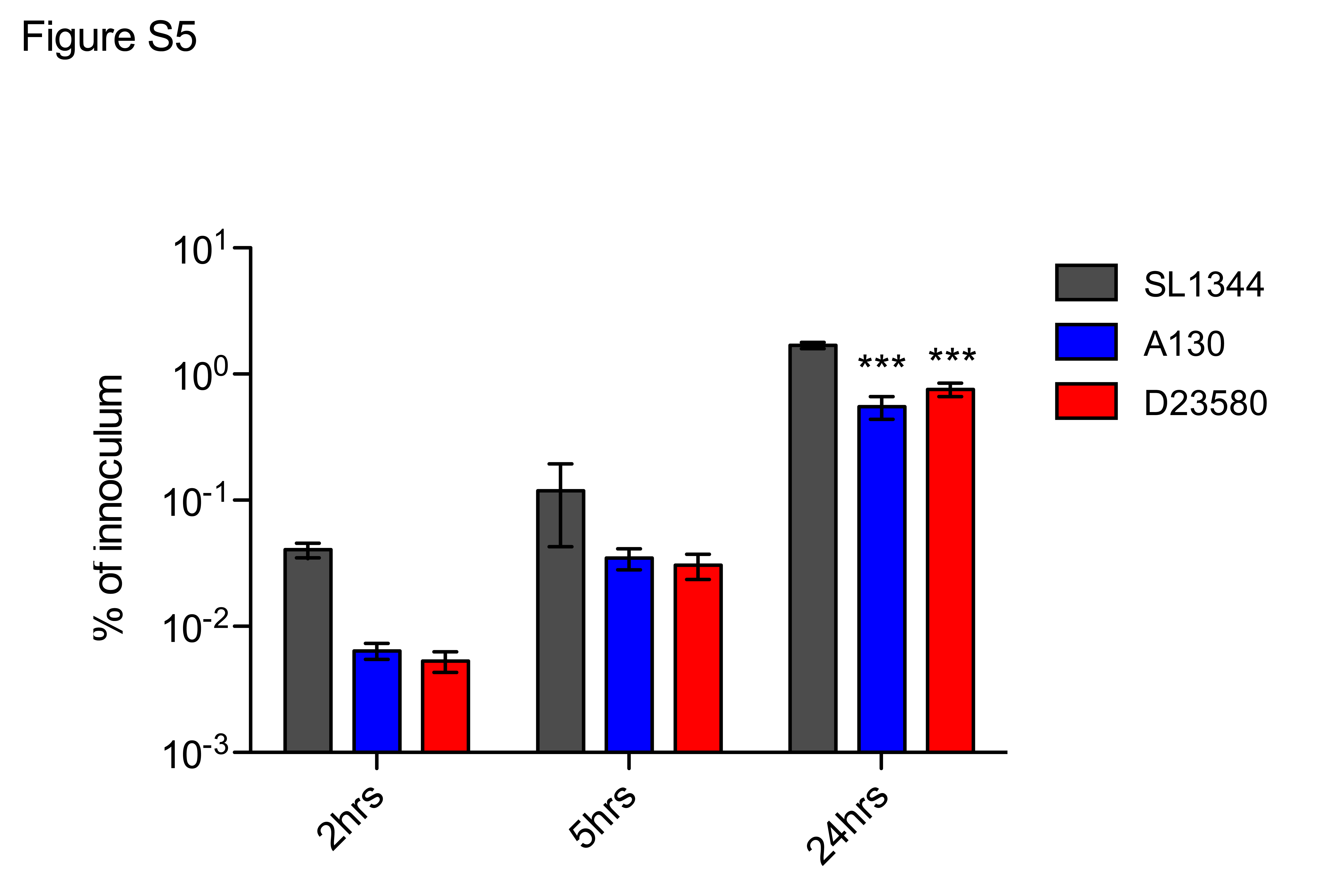

Supplement: S5 Fig — Asterisks indicate statistically significant differences at p < 0.001 using a two-way ANOVA with post-tests performed using the Bonferroni method. (TIF) [file pntd.0003611.s006.tif]
